# Thermally Regulated Droplet Microfluidics Enables Uniform, Size‐Controllable DNA‐Nanoparticle Superlattices

**DOI:** 10.1002/smll.74787

**Published:** 2026-08-10

**Authors:** Naotomo Tottori, Miho Tagawa, Azusa Takao, Lidong Zhang, Maasa Yokomori, Shinya Sakuma, Yoko Yamanishi

**Affiliations:** ^1^ Department of Mechanical Engineering Faculty of Engineering Kyushu University Fukuoka Japan; ^2^ Division of Materials Research (DM) Institute of Materials and Systems for Sustainability (IMaSS) Nagoya University Nagoya Japan; ^3^ Department of Materials Process Engineering Nagoya University Nagoya Japan; ^4^ Department of Precision Mechanical Engineering School of Science and Engineering Chuo University Tokyo Japan

**Keywords:** confinement, DNA‐functionalized nanoparticle superlattices, droplet microfluidics, single crystallite, thermal regulation

## Abstract

DNA‐functionalized nanoparticles (DNA‐NPs) can self‐assemble into superlattices with unique optical and electronic properties, and their ability to encapsulate macromolecular cargo makes them promising carriers for molecular delivery. To support such applications, crystals with uniform size and high lattice order are required. However, conventional batch crystallization can be affected by heterogeneous nucleation, convection, and aggregation, leading to broad size distributions and poor structural order. In this paper, we present a thermally regulated droplet‐microfluidic strategy that produces uniform, size‐controllable, single‐crystallite DNA‐functionalized gold nanoparticles (DNA‐AuNPs) superlattices in monodisperse water‐in‐oil droplets. Droplets are produced at 65°C to suppress premature hybridization, and then ultra‐slowly cooled to promote near‐equilibrium assembly. We identify a predominantly single‐crystallite regime in smaller droplets (≈9–23 µm) and achieve a ninefold reduction in size variability compared to batch processing.  Small‐angle X‐ray scattering analysis indicates that the characteristic domain size remains approximately constant across droplet sizes, suggesting that differences in crystallization behavior arise from size‐dependent nucleation statistics rather than intrinsic improvements in crystal quality. Confinement may suppress multi‐nucleation and convective perturbations, potentially providing a route to DNA‐AuNP superlattices with controllable size, number, and order under confined conditions, which could enable dose‐quantized delivery and applications in plasmonic and electronic materials.

## Graphical abstract

Thermally regulated droplet microfluidics enables controlled assembly of DNA‐functionalized gold nanoparticles into uniform, size‐controllable superlattice crystals. Monodisperse droplets formed at elevated temperature suppress premature hybridization, followed by ultra‐slow cooling to promote near‐equilibrium growth. Confinement limits multiple nucleation, yielding predominantly single‐crystallite structures and significantly reduced size variability, enabling reproducible nanostructure fabrication.

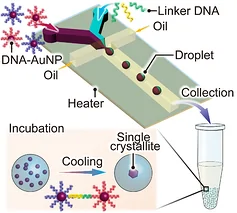

## 1 Introduction

DNA‐functionalized nanoparticles (DNA‐NPs) exploit sequence‐programmable hybridization to self‐assemble into ordered superlattices with precisely tunable interparticle spacing and symmetry [1, 2, 3, 4], thereby enabling emergent optical and electronic properties that are inaccessible to isolated nanoparticles or bulk materials [5, 6]. These emergent properties have positioned DNA‐NP superlattices as promising building blocks for advanced plasmonic and electronic devices, including plasmonic sensors, surface‐enhanced Raman scattering (SERS) substrates, and metamaterials for light manipulation, enabled by their precisely engineered lattice architecture [5, 7]. Beyond optical and electronic applications, DNA‐NP assemblies offer unique opportunities in biotechnology. When organized into porous superlattices, these crystals can encapsulate macromolecules such as proteins and CRISPR/Cas9 ribonucleoproteins, enabling direct intracellular delivery via particle bombardment [8]. This capability establishes DNA‐NP crystals as cargo‐capable carriers for molecular delivery and, in principle, dose‐quantized vectors—provided that crystal size and structural order can be precisely controlled.

Despite these advances, achieving precise control over crystal size and structural order remains challenging. Traditionally, DNA‐NP crystals have been produced by batch crystallization in microtubes, a process that relies on slow thermal annealing to drive hybridization‐mediated assembly. However, batch methods may be less suitable for producing large populations of discrete DNA‐NP crystals with controlled number, size, and minimal aggregation. In such systems, heterogeneous nucleation at container walls or impurities, convective transport during growth, and inter‐crystal aggregation can introduce variability in nucleation and growth processes [8, 9]. These factors may broaden crystal size distributions and complicate control over structural uniformity, which can impact dose precision for delivery applications and hinder integration into plasmonic or electronic devices [8]. These limitations are particularly relevant when precise control over individual crystal size and population uniformity is required. Furthermore, batch crystallization also offers limited scalability and reproducibility, as subtle variations in thermal gradients or solution conditions can significantly alter nucleation kinetics. Collectively, these limitations highlight the need for strategies that provide precise control over nucleation and growth while maintaining uniformity across large populations of crystals.

Droplet microfluidics has emerged as a powerful approach for addressing similar challenges in other colloidal and biomolecular systems [10, 11, 12, 13, 14, 15]. By generating monodisperse, well‐defined microreactors, droplet platforms can suppress convection and isolate nucleation events, enabling improved control over crystallization kinetics. These advantages have been leveraged to study protein crystallization and colloidal assembly, where droplet confinement minimizes heterogeneous nucleation and promotes uniform growth [16, 17, 18, 19, 20, 21]. For DNA‐coated colloids at the submicrometer scale (∼600 nm), droplet‐based studies have elucidated nucleation and growth dynamics, revealing that classical nucleation theory must be modified to account for friction‐limited bond rearrangements at crystal interfaces [22, 23]. Despite these advances, to the best of our knowledge, droplet microfluidics has not yet been applied to produce uniform, highly ordered superlattices from nanoscale (∼10 nm) DNA‐NPs. While previous studies have demonstrated confinement‐mediated assembly in DNA‐NP systems [24], extending such approaches to thermally regulated microfluidic droplets at the nanoscale remains challenging. This gap is significant, as nanoscale DNA‐NP crystals are essential for molecular delivery, plasmonic metamaterials, and quantum‐scale electronic architectures. However, achieving defect‐minimized, size‐controllable crystals at this scale remains a key challenge.

To address this limitation, we introduce a thermally regulated droplet microfluidic strategy for producing highly uniform, size‐controllable single‐crystallite DNA‐functionalized gold nanoparticle (DNA‐AuNP) superlattices. Our approach employs monodisperse water‐in‐oil (W/O) microdroplets as isolated reaction vessels, generated under elevated temperature and subsequently annealed by slow cooling to promote controlled self‐assembly. Using the microfluidic system, we reproducibly achieve superlattice formation within each droplet, identify a single‐crystallite regime, predominantly in droplets smaller than ∼23 µm, and demonstrate a ninefold reduction in size variability compared with batch crystallization (coefficient of variation (CV) reduced from 37% to 4%). Here, “single‐crystallite” refers to a single contiguous superlattice object observed within a droplet, without implying strict crystallographic single crystallinity. Small‐angle X‐ray scattering (SAXS) analysis suggests that the characteristic domain size remains approximately constant across droplet sizes, indicating that lattice coherence is largely independent of droplet size within the present experimental conditions. To interpret these results appropriately, we note that the present SAXS measurements are ensemble‐averaged and therefore do not resolve internal domain structures at the single‐object level. Collectively, these findings suggest that microdroplet confinement may provide a robust and scalable method for producing size‐controllable, cargo‐capable DNA‐NP crystals, potentially enabling new opportunities for dose‐quantized molecular delivery and advancing potential applications in plasmonic and electronic materials.

## 2 Results

### 2.1 Outline of the Constructed System

**FIGURE 1:**
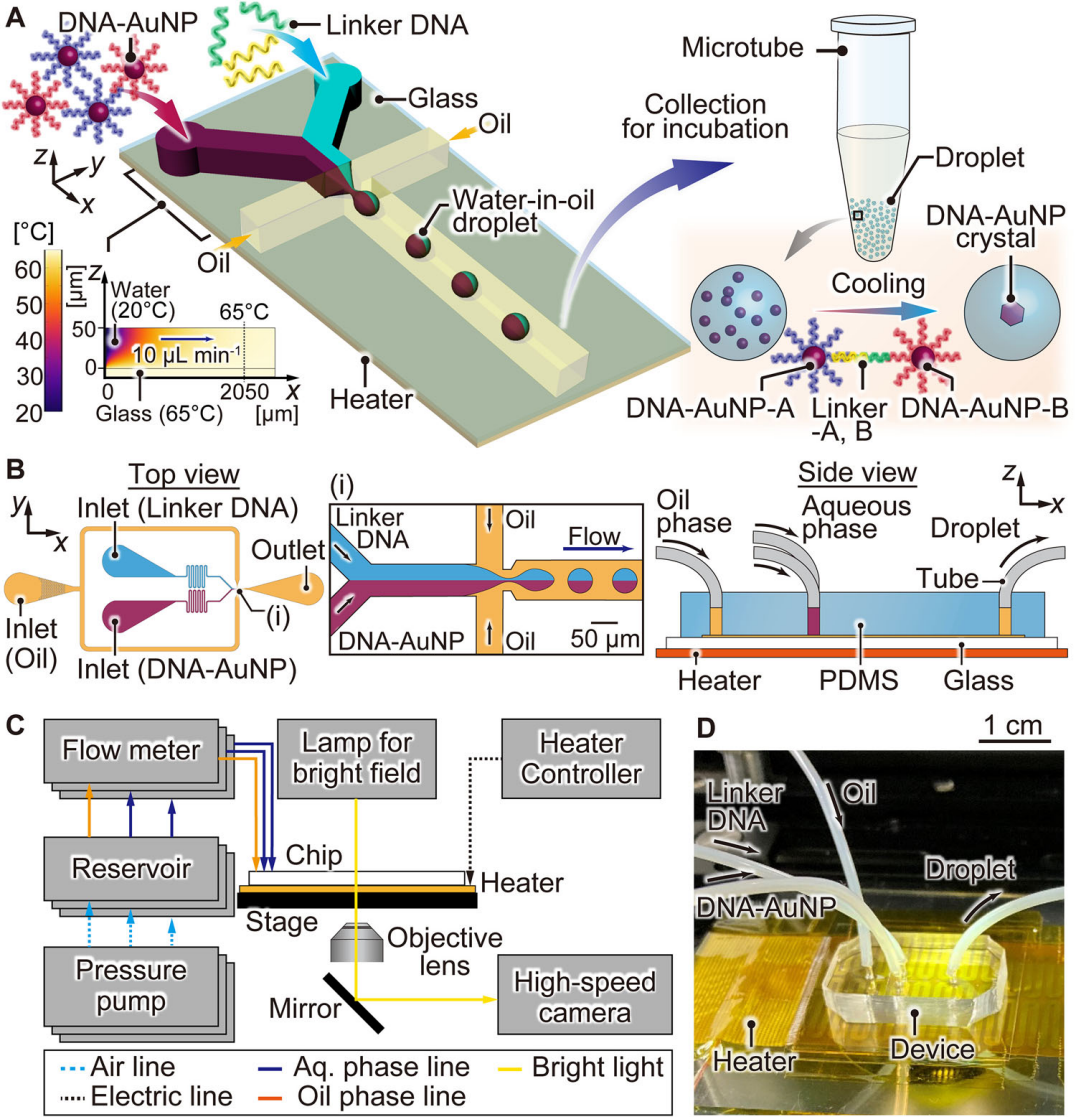
Thermally regulated droplet‐microfluidic platform for DNA‐AuNP crystallization. (A) Integrated workflow: on‐chip droplet generation at 65°C, off‐chip ultra‐slow cooling (from 55°C to 35°C, 0.01°C min^−^^1^) for DNA‐AuNP crystallization. (B) Microfluidic chip with three inlets (two aqueous, one oil), a flow‐focusing cross‐junction, and a single outlet; inset shows the junction geometry. (C) System‐level schematic (chip, film heater with closed‐loop control, pressure‐driven fluid delivery, optical observation). (D) Photograph of the fabricated microfluidic device mounted on a film heater.

The proposed microfluidic platform (Figure 1A) integrates droplet generation with thermal regulation and off‐chip crystallization into a single workflow. Two aqueous streams—DNA‐NP solution and linker DNA solution (Figure S1)—merge in a Y‐shaped channel and pass through a serpentine preheating section before emulsification at a flow‐focusing junction (Figure 1B). Monodisperse W/O droplets are generated under thermal control (∼65°C) to prevent premature hybridization and aggregation, with size tuned by adjusting flow rates and orifice width (20 or 40 µm). After generation, droplets are collected in microtubes and subjected to ultra‐slow cooling (from 55°C to 35°C at 0.01°C min^−^^1^) to promote near‐equilibrium DNA hybridization and ordered crystal growth (Figure 1A). The microfluidic chip for droplet generation is integrated into the overall system (Figure 1C), with an enlarged view of the chip shown in Figure 1D.

### 2.2 Formation of DNA‐AuNP Encapsulated Droplet

**FIGURE 2:**
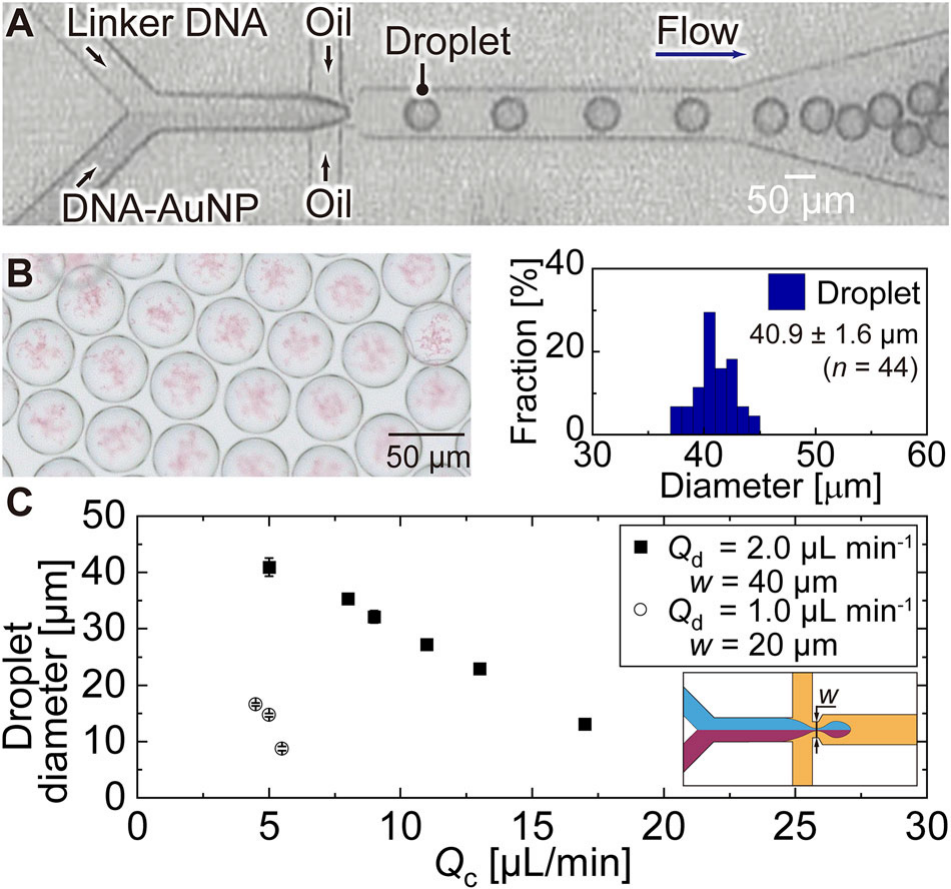
Formation of DNA‐AuNP‐encapsulated microdroplets. (A) Dripping‐regime droplet formation at the cross‐junction at 65°C (*Q*_c_ = 5.0 µL min^−1^ and *Q*_d_ = 2.0 µL min^−1^, composed of two equal streams of 1.0 µL min^−1^ each). (B) Collected droplets with the corresponding size‐distribution histogram (mean ± s.d., *n* = 44 droplets). (C) Variation of droplet diameter as a function of *Q*_c_ with fixed *Q*_d_ at 2.0 µL min^−1^ (40 µm orifice) and 1.0 µL min^−1^ (20 µm orifice). Each data point represents the mean droplet diameter; sample sizes correspond to those reported in Figure 3 (typically *n* ≈ 40–320), except for *Q*_c_ = 5 µL min^−^^1^ (*D*_d_ ≈ 40.9 µm), where *n* = 44.

Uniform W/O droplets containing DNA‐AuNPs were reproducibly generated at a flow‐focusing microfluidic cross‐junction under controlled heating at 65°C. Heating was essential to maintain stable two‐phase laminar flow and to suppress premature interfacial aggregation between DNA‐AuNP and linker streams. Without heating, interfacial aggregation caused unstable breakup and irregular droplet formation (Figure S2). Thermal simulations of the serpentine pre‐heating section confirmed that the centerline fluid reaches ∼65°C within ∼2.0 mm under our conditions (Figure S3), ensuring on‐chip pre‐equilibration before emulsification. Under these conditions, monodisperse droplets formed consistently across a specific range of continuous (*Q*_c_​) and dispersed (*Q*_d_​) phase flow rates and were collected in microtubes. At *Q*_c_ = 5.0 µL min^−1^ and *Q*_d_ = 2.0 µL min^−1^ (1.0 µL min^−1^ × 2), droplets were generated in a dripping regime at approximately 597 drops s^−1^ (Figure 2A), producing droplets with a mean diameter of 40.9 ± 1.6 µm (standard deviation (s.d.)) (Figure 2B).

We next examined the effect of flow rates and orifice geometry on droplet diameter using devices with 20 and 40 µm orifices (Figure 2C). For the 20 µm orifice, *Q*_d_ was fixed at 1.0 µL min^−1^ (0.5 µL min^−1^ × 2) while *Q*_c_ was varied from 4.5 to 5.5 µL min^−1^, producing droplets with diameters ranging from 8.8 to 16.6 µm. For the 40 µm orifice, *Q*_d_ was fixed at 2.0 µL min^−1^ while *Q*_c_ was varied from 5.0 to 17.0 µL min^−1^, producing droplets with diameters ranging from 13.1 to 40.9 µm. Increased *Q*_c_ reduced droplet size due to high shear, and the 20 µm orifice—imparting higher local shear than the 40 µm orifice—produced smaller droplets across the entire tested range, including at low *Q*_c_​ conditions. Under these tested conditions, droplet diameters were controllably tuned from 8.8 to 40.9 µm by selecting the orifice size (Figure 2C). Thus, the droplet size range investigated throughout this study spans approximately 9–41 µm.

To support that mixing inside the droplets is diffusion‐dominated under our 65°C conditions, we estimate a characteristic diffusion time using the standard scaling *τ ≈ L* ^2^/*D* [25]. Hence, *D* is the translational diffusion coefficient of the DNA‐NPs, *L* is the droplet radius used as the characteristic mixing length, and the value of *D* is obtained from the Stokes–Einstein relation (*D* = *k*_B_ *T*/6π*ηr*) with the Boltzmann constant (*k*_B_), the absolute temperature (*T*), the dynamic viscosity of the medium (*η*), and the hydrodynamic radius of the nanoparticle (*r*). Taking *L* as the droplet radius for typical single‐crystal droplets (droplet diameter (*D*_d_) *≈* 20 µm, hence *L ≈* 10 µm) and diffusion coefficient (*D*) *≈* 1.1 × 10^−10^ m^2^ s^−1^ for ∼10 nm DNA‐NPs at 338 K, we obtain *τ ≈* 0.9 s, consistent with rapid homogenization (Figure S4).

### 2.3 DNA‐AuNP Crystallization in Droplets

**FIGURE 3:**
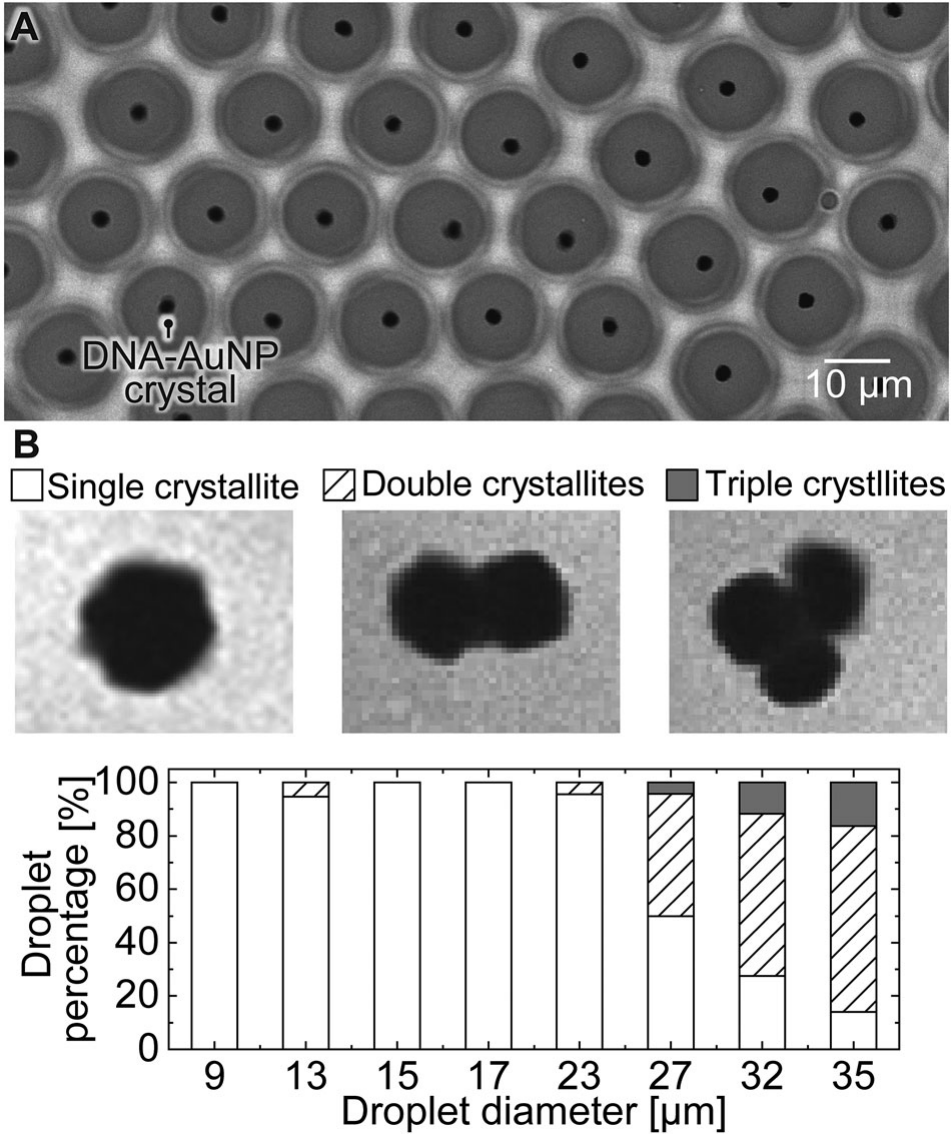
Size‐dependent crystal multiplicity in microdroplets. (A) Collected droplets each containing a single crystallite. (*Q*_c_ = 4.5 µL min^−1^ and *Q*_d_ = 1.0 µL min^−1^ (0.5 µLmin^−1^ × 2)). (B) Examples of single, double, and triple crystallites, and the percentage of droplets containing each crystal number as a function of droplet diameter. The number of droplets analyzed for each diameter is as follows: 9 µm (*n* = 320), 13 µm (*n* = 75), 15 µm (*n* = 103), 17 µm (*n* = 87), 23 µm (*n* = 45), 27 µm (*n* = 94), 32 µm (*n* = 102), and 35 µm (*n* = 43).

After incubation under controlled cooling (from 55°C to 35°C at 0.01 °C min^−1^), DNA‐AuNP crystals were observed within the droplets. Figure 3A shows a representative example of DNA‐AuNP crystallization in droplets (mean diameter: 16.6 µm) generated at *Q*_c_ = 4.5 µL min^−1^ and *Q*_d_ = 1.0 µL min^−1^ (0.5 µL min^−1^ × 2). Crystals formed consistently across the produced droplets. Analysis of droplet diameter versus crystal number revealed a clear size‐dependent trend: droplets 9–23 µm in diameter predominantly contained a single‐crystallite object, whereas droplets larger than 23 µm increasingly exhibited doublets or triplets, remaining at ∼5% up to 23 µm and rising to ∼50% at 27 µm (Figure 3B). Notably, the crystallization yield—defined as the fraction of droplets containing at least one crystal—was effectively quantitative under our operating conditions, with nearly all droplets producing crystals.

**FIGURE 4:**
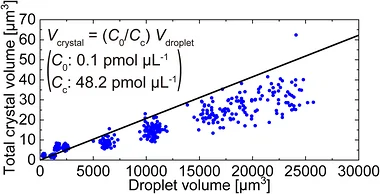
Scaling of total crystal volume with droplet volume. Variation of total crystal volume (*V*_crystal_) as a function of droplet volume (*V*_droplet_). The solid line represents the mass‐balance prediction, *V*_c_ = (*C*_0_/*C*_c_) *V*_d_, using the DNA‐AuNP concentration in the droplet (*C*_0_ = 0.10 pmol µL^−^^1^) and the DNA‐AuNP number density in the crystal (*C*_c_ = 48.2 pmol µL^−^^1^) estimated for a bcc superlattice *a* = 41 nm. Points are experimental data. A linear fit is shown for droplets with a diameter *D* < 17 µm (*R* ^2^ = 0.89). Each data point corresponds to the droplet populations shown in Figure 3, where the sample sizes (*n*) for each condition are provided.

The total crystal volume per droplet (sum of all crystals within a droplet) was plotted against the corresponding droplet volume (Figure 4). As droplet size increased, the total crystal volume also increased, indicating that the amount of crystallized material scales with the available volume. This relationship can be expressed as:

(1) $$V_{\mathrm{crystal}}=\frac{C_{0}}{C_{c}}V_{\mathrm{droplet}}$$

where *C*_0_​ is the DNA‐AuNP concentration in the droplet (pmol µL^−^^1^), *C*_c_​ is the DNA‐AuNP number density in the crystal (pmol µL^−^^1^), *V*_crystal_​ is the total crystal volume, and *V*_droplet_​ is the droplet volume. Using *C*_0_ = 0.1 pmol µL^−1^ and *C*_c_ = 48.2 pmol µL^−1^ (estimated for a body‐centered cubic (bcc) superlattice with a 41 nm lattice constant), a theoretical line was calculated and overlaid on the experimental data. The observed data for smaller droplets (<17 µm) fit the theoretical prediction well (R^2^ = 0.89), indicating efficient incorporation of encapsulated DNA‐AuNPs into crystals.

### 2.4 Comparison With Batch Crystallization

**FIGURE 5:**
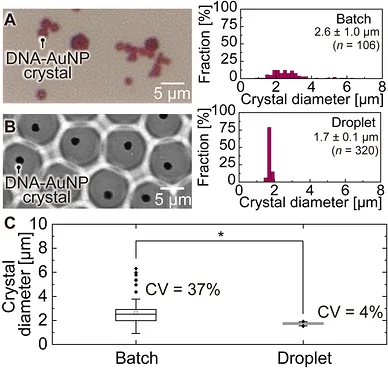
DNA‐AuNP colloidal crystals produced by batch vs droplet methods. (A) Photomicrographs of crystals produced by the batch method and (B) the droplet method, with corresponding size‐distribution histograms (mean ± s.d.). *n* indicates the number of crystals analyzed for each condition. (C) Box‐and‐whisker plot comparing crystal‐diameter distributions for two methods, with the coefficient of variation (CV) indicated. Boxes denote the interquartile range (IQR) with the median line; whiskers extend to the most extreme data points within 1.5 × IQR, and points beyond are plotted as outliers. The asterisk (*) indicates *p* < 0.05 (two‐sided Welch's t‐test) for the difference in mean crystal diameter between batch and droplet samples.

To evaluate the advantage of droplet‐based crystallization, we compared it with the batch technique performed in microtubes (Figure 5). The data shown represent a representative dataset from independent experiments exhibiting consistent trends. In the batch process, crystals of varying sizes were observed, along with noticeable aggregation (Figure 5A). The measured crystal equivalent diameter (*D*_hex_), estimated from a hexagonal projection of a rhombic dodecahedron, averaged 2.6 µm with a coefficient of variation (CV) of 37%. In contrast, crystals formed within droplets (mean droplet diameter: 16.6 µm, generated at *Q*_c_ = 5.5 µL min^−^^1^ and *Q*_d_ = 1.0 µL min^−^^1^ (0.5 µL min^−1^ × 2)) exhibited uniform sizes (Figure 5B), with an average *D*_hex_ of 1.7 µm and a CV of 4%. While the average crystal size differs between the two methods, the most notable difference lies in the substantial reduction in size variability achieved in the droplet‐based system. These results demonstrate a ninefold reduction in size variability in the droplet‐based method compared to batch crystallization (Figure 5C). The statistical significance of the difference in mean crystal size was evaluated using a two‐sided Welch's t‐test (*p* < 0.05), as indicated in Figure 5C.

### 2.5 Crystal Quality Evaluation

**FIGURE 6:**
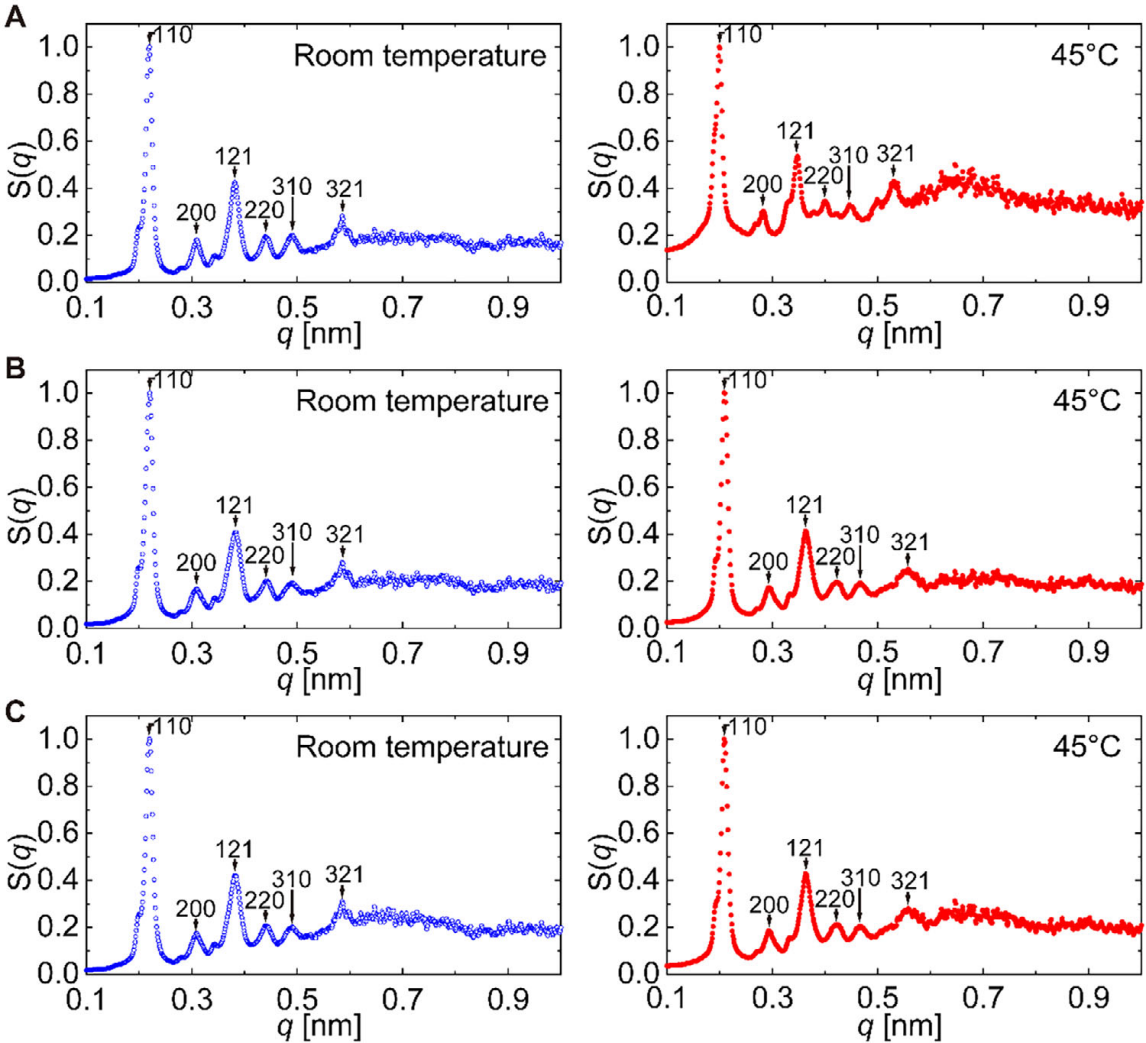
SAXS analysis of DNA‐AuNP superlattices formed in droplets of different sizes. SAXS intensity profiles of DNA‐AuNP superlattices formed in droplets with mean diameters (A) 15 µm, (B) 21 µm, and (C) 46 µm recorded at room temperature and at 45°C.

To assess the lattice quality of the DNA‐AuNP crystals formed in microdroplets, small‐angle X‐ray scattering (SAXS) analysis was conducted. Crystals formed in droplets with mean diameters of 15, 21, and 46 µm were compared. By analyzing the peak position ratios in structure factors *S(q)*, we confirmed that the DNA‐AuNP crystals, assembled in droplets of any size, had bcc structures as designed. Crystallographic analysis revealed that the lattice constants of the hydrated DNA‐AuNP crystals are 40.7, 40.5, and 40.7 nm at room temperature, respectively, and were nearly identical. The *g*‐factor, which is an index of structural coherence derived from the full width at half maximum (FWHM) of the first peak in the SAXS profile [26, 27], was found to be 0.060, 0.065, and 0.065 for crystals grown in droplets of 15, 21, and 46 µm diameter, respectively. Since differences in the internal structure of the crystals may affect the coefficient of thermal expansion of the crystals during heating, each DNA‐AuNP crystal sample was gradually heated from room temperature (25°C) to 45°C at a rate of 2°C every 3 min and equilibrated at 45°C prior to SAXS measurement. In contrast to room temperature, at 45°C, the lattice constants of the hydrated DNA‐AuNP crystals increased to 44.8, 42.8, and 42.8 nm, respectively, with a somewhat larger increase in the lattice constant observed for crystals assembled within droplets of 15 µm diameter (Figure 6). Although the g‐factors differed only slightly among the samples, differences were observed in the increase in lattice constants at 45°C. The increase in lattice constants was greatest for crystals assembled within droplets of 15 µm diameter, suggesting possible differences in thermal expansion behavior.

In some profiles, weak shoulders or satellite‐like features are observed, which may reflect lattice distortion, strain, or the coexistence of multiple lattice populations; however, these features are not quantitatively analyzed due to the ensemble‐averaged nature of the SAXS measurements. The characteristic domain size estimated from SAXS remains approximately constant (∼400 nm) across droplet sizes. Additional details on the peak profile analysis are provided in the Supporting Information (see ESI, Section S1). It should be noted that these SAXS measurements represent ensemble‐averaged data over multiple superlattice objects and therefore do not resolve internal domain boundaries or crystallographic defects at the single‐object level. Accordingly, even a droplet containing a single observed object may actually consist of multiple domains, and the possibility that it consists of aggregates of independently formed crystals cannot be ruled out.

## 3 Discussion

### 3.1 Mechanistic Basis for Improved Uniformity and Suppression of Batch Limitations

Microdroplet confinement is proposed to provide a controlled microenvironment that may mitigate key limitations of batch crystallization. Physical isolation from container walls may reduce heterogeneous nucleation, while low‐Reynolds‐number conditions can suppress bulk convection and favor diffusion‐dominated transport, potentially promoting more uniform supersaturation during growth. Consistent with these effects, droplets produced predominantly single‐crystallite objects below ∼23 µm and displayed markedly lower size variability than batch (approximately ninefold reduction in CV under our conditions). The approximately linear scaling of crystal volume with droplet volume further aligns with a mass‐balance‐limited growth regime rather than being strongly limited by slow molecular processes, as supported by Figure 4 showing a near‐linear *V*_crystal_–*V*_droplet_ relationship. Extending droplet‐based crystallization beyond submicron colloids, our platform produces nanoscale DNA‐NP crystals with controllable size and well‐defined lattice structure, as characterized by SAXS.

Three droplet‐enabled features may plausibly contribute to the observed gains in uniformity and size control. (i) Reduced wall‐mediated heterogeneous nucleation: Oil‐separated aqueous droplets physically isolate the crystallization environment and minimize interaction with solid boundaries and container‐borne/adventitious nucleants, thereby lowering the probability of wall‐triggered nucleation events [15].  (ii) Suppression of convection during crystal growth: At the microdroplet scale under low‐Reynolds‐number conditions, internal recirculation is reduced, favoring diffusion‐based transport and more homogeneous solute fields around growing crystals. Marangoni flows—driven by interfacial tension gradients—may also be weaker in small droplets because thermal and solutal gradients equilibrate rapidly at this scale [20].  This is consistent with the observed approximately linear relationship between crystal and droplet volumes and with the prevalence of single‐crystallite outcomes in smaller droplets, where weaker convective recirculation reduces local supersaturation fluctuations during nucleation and growth. (iii) Suppression of crystal–crystal collisions: Encapsulation limits occupancy to one or a few crystals per droplet, and surfactant stabilization prevents inter‐droplet coalescence, removing a common post‐nucleation aggregation pathway in batch systems. Collectively, these effects likely narrow the nucleation window and homogenize growth conditions, explaining the lower CV and higher fraction of single‐crystallite outcomes. These mechanisms are proposed as plausible explanations consistent with the observed trends, rather than being directly demonstrated in the present study.

### 3.2 Impact of Confinement on Crystal Quality

The size‐dependent transition from predominantly single‐crystallite objects in smaller droplets to multi‐domain structures in larger droplets can be understood in terms of stochastic nucleation probabilities, as described by classical nucleation theory. Detailed theoretical considerations are provided in the Supporting Information (see ESI, Section S2). This statistical framework is consistent with the experimentally observed trends in crystal multiplicity. In smaller droplets, the probability of multiple nucleation events is reduced, favoring the formation of predominantly single‐crystallite objects. In contrast, larger droplets can accommodate multiple nucleation events, resulting in multi‐domain or multiplet structures. We therefore interpret the observed transition around ∼20–25 µm in terms of a critical droplet‐size regime governing the probability of single‐domain formation under confinement. Consistent with this interpretation, the SAXS results show only minor variations in scattering intensity and g‐factor values across droplet sizes, suggesting that while nucleation statistics affect crystal multiplicity, they do not strongly alter the intrinsic lattice coherence within individual crystals. While SAXS profiles show slightly higher scattering intensity and lower g‐factor values for crystals grown in ≈15 µm droplets, these differences are small and do not support a strong claim of intrinsically improved crystal quality. Instead, the SAXS‐derived characteristic domain size remains approximately constant across droplet sizes, suggesting that lattice coherence is largely independent of droplet size within the present experimental conditions. These observations indicate that the apparent differences arise primarily from size‐dependent nucleation statistics rather than intrinsic improvements in crystal quality. The slightly reduced peak splitting observed in ∼15 µm droplets may reflect reduced heterogeneity in lattice constants; however, this effect is considered secondary and not sufficient to explain the overall trends in crystallinity.

Further structural characterization using complementary high‐resolution imaging techniques such as TEM or STEM would provide additional insight into internal crystallographic domain structure within the superlattices. Previous studies have shown that sample preparation for TEM (e.g., drying or resin embedding) can introduce lattice deformation and partial disordering, as well as changes in lattice parameters compared with in situ SAXS measurements [4]. Therefore, while TEM and STEM are valuable complementary techniques, their results should be interpreted with consideration of preparation‐induced artifacts when comparing to hydrated structures in solution. Investigation of internal domain structure in situ remains an important direction for future work.

### 3.3 Technical Considerations, Manufacturing Strategy, and Future Directions

#### 3.3.1 Technical Considerations

Maintaining an ultra‐slow cooling rate (0.01°C min^−1^) is central to near‐equilibrium assembly [27]. Accordingly, both on‐chip thermal lanes and off‐chip microtube cooling should exhibit narrow residence‐time distributions and minimal thermal gradients to avoid broadening the supersaturation windows and triggering parallel nucleation. In parallel, surfactant/oil selection (e.g., Isopropyl palmitate with ABIL EM 180) governs interfacial stability, Marangoni flows, and coalescence resistance; since surfactants can alter the effective interfacial energy γ—and thus the barrier Δ*G* ^∗^—(where γ is the effective interfacial energy and Δ*G* ^∗^ is the nucleation barrier; see ESI, Section S2), their impact warrants quantitative assessment. Additionally, ionic strength and linker stoichiometry determine screening and hybridization kinetics, thereby shifting Δ*µ* and the effective kinetic prefactor *J*_0_^(eff)^ (where Δ*µ* is the supersaturation driving force and *J*_0_^(eff)^ is the effective kinetic prefactor incorporating rearrangement‐limited kinetics; see ESI, Section S2); standard operating ranges should be defined with tolerance bands to keep the process within the single‐crystal window. For metrology, crystal measurements should minimize biases from 2D projections‐ multi‐plane imaging or light‐sheet microscopy can improve crystal counting and volumetric accuracy, especially in larger droplets. Finally, during post‐processing, emulsions should remain stabilized during transfer and up to crystal isolation to suppress Ostwald ripening and collision‐mediated aggregation; short residence times and gentle handling further reduce risk.

#### 3.3.2 Manufacturing Strategy of DNA‐NP Crystals

The microdroplet platform may support high‐throughput production through numbering‐up and continuous operation, while preserving crystal quality via in‐line monitoring and tight thermal control. Parallelization of flow‐focusing junctions [28, 29]—or, alternatively, arrays of step‐emulsification nozzles [30, 31]—on a single chip (or across multiple chips) increases the droplet generation rate approximately linearly with the number of junctions/nozzles and maintains monodispersity under identical hydraulic and thermal conditions; step‐emulsification, in particular, achieves geometry‐set droplet sizes at low capillary numbers and can be massively parallelized via nozzle arrays. Continuous production could be realized by integrating (i) on‐chip emulsification at elevated temperature (e.g., 65°C) to stabilize two‐phase flow and prevent premature interfacial aggregation of DNA‐NPs and linkers, thereby potentially ensuring uniform encapsulation and run‐to‐run reproducibility; (ii) in‐line thermal annealing through serial temperature zones (e.g., an isothermal hold at 55°C followed by ultra‐slow ramps to 35°C), to reproducibly access the nucleation/growth regime across droplet diameters; and (iii) off‐chip collection with surfactant‐stabilized emulsions to prevent coalescence. A modular design—chip cartridges plus thermal lanes—may facilitate maintenance and parallel expansion without perturbing local hydrodynamics, thereby potentially supporting reproducible large‐scale fabrication. Quality assurance may be achieved by real‐time in‐line optical monitoring of droplet diameter and loading (occupancy), followed by post‐annealing quality checks‐ either high‐throughput microscopy to quantify per‐droplet crystal occupancy or batch SAXS to assess lattice order. Critically, scale‐up would require maintaining the droplet‐diameter distribution (via fixed orifice, closed‐loop pressure/flow control) and careful control of cooling profiles (closed‐loop thermal control, calibrated residence times), thereby helping to preserve the single‐nucleation window and confinement‐induced ordering identified in small droplets.

#### 3.3.3 Future Directions

First, systematically establish operating phase maps across droplet diameter, cooling rate, and composition, and overlay quality isoclines (SAXS FWHM, correlation length, higher‐order features) to create an actionable process design map that delineates single‐crystal domains and guides dose‐quantized production by balancing mass (via diameter selection) with nucleation control (via thermal schedules). Next, quantify kinetics by fitting multiplicity statistics to *P*(≥*n*) models under controlled thermal schedules and extract effective parameters such as *J*_0_^(eff)^ and Δ*G* ^∗^; compare these with friction‐limited nucleation frameworks for DNA‐coated colloids to clarify how Δ*µ* and the rearrangement timescale *τ*_rearr_​ regulate nucleation windows and crystalline order (*τ*_rearr_ represents the interfacial rearrangement timescale governing defect‐minimized growth; see ESI, Section S2). Additionally, expand sequence and lattice engineering—vary spacer lengths and linker designs—to tune lattice constants and binding kinetics, thereby probing how Δ*µ* and *τ*_rearr_ control order across compositions. In parallel, develop post‐growth solution‐injection strategies: (a) feed additional DNA‐NPs to enable epitaxial crystal enlargement without compromising order, and (b) sequentially introduce distinct linkers or particle species to realize heterostructures (core–shell or concentric superlattices) via selective hybridization. These hierarchical assemblies could broaden functionality toward multi‐modal plasmonic/electronic materials and programmable cargo encapsulation. Finally, given the inherent reproducibility and throughput of droplet microfluidics, the approach may be well‐positioned for scale‐up, on‐chip screening, and integration with downstream assembly or delivery modules. Future work should align process control (locking diameter distributions and standardizing cooling profiles) with in‐line quality monitoring to ensure robust, reproducible production runs, ultimately enabling potential application‐oriented targets—for example, dose‐quantized intracellular delivery, SERS/metamaterial platforms with tighter lattice order, and quantized optical/electronic devices built from DNA‐NP superlattices.

## 4 Conclusion

We demonstrated a droplet‐microfluidic strategy to produce uniform, size‐controllable DNA‐NP crystals under controlled thermal conditions. By generating monodisperse W/O droplets at 65°C and applying slow, controlled cooling (from 55°C to 35°C at 0.01°C min^−^^1^), we reproducibly formed uniform crystals whose total crystal volume scaled linearly with droplet volume in accordance with a mass‐balance model. We also mapped the size‐dependent crystal multiplicity, identifying a predominantly single‐crystallite regime in smaller droplets (typically 9–23 µm), with multiplets becoming more frequent at larger diameters. Compared with batch crystallization in microtubes, the droplet process reduced size dispersion nine‐fold (CV 4% compared to 37%) while maintaining comparable mean sizes and minimizing aggregation. SAXS measurements further suggest that the characteristic domain size remains approximately constant across droplet sizes, indicating that the observed differences arise primarily from size‐dependent nucleation statistics rather than intrinsic improvements in crystal quality. Overall, these results suggest that microdroplet confinement may provide a robust and potentially scalable route to crystals with controllable size and number, which could enable dose‐quantized production for cargo delivery and plasmonic/electronic metamaterials.

## 5 Experimental Section

### 5.1 Microfluidic Device Design

We designed microfluidic devices that comprise three inlets–two for aqueous phases (DNA‐AuNP solution and linker DNA solution) and one for the oil phase–a flow‐focusing cross‐junction for droplet generation, and a single outlet for droplet collection in a microtube (Figure 1). A filter structure was incorporated at the continuous phase inlet to remove particulates that could disrupt droplet formation. Upstream of the junction, the two aqueous streams merged in a Y‐shaped channel and then traversed a serpentine preheating section before emulsification (Figure 1B). Two device geometries were used: one with a 50 µm channel depth and a 40 µm orifice, and another with a 25 µm depth and a 20 µm orifice. These dimensions were determined to minimize droplet flattening and coalescence while targeting spherical W/O droplets in the 10–40 µm range. At the droplet generation region, channel widths for the aqueous and oil inlets were 50 µm, and the outlet (drain channel) was 75 µm in both device designs (Figure 1B inset). These widths set the local shear and capillary number at the junction, enabling stable formation of monodisperse droplets over the desired size window.

On‐chip thermal control was implemented with a film heater placed beneath the microchannel, maintaining ∼65°C during droplet formation. This elevated pre‐emulsification temperature kept DNA bridges dissociated so that DNA‐AuNPs remained fully dispersed, preventing premature aggregation where the linker and DNA‐AuNP streams met. The sufficient serpentine length (∼2.0 mm) was determined from thermal simulations to allow an inflow at 20°C to reach ∼65°C at a 10 µL min^−^^1^ flow in 50 × 50 µm channels (Figure S3). Beyond thermal equilibration, the serpentine also increased the hydraulic resistance of the aqueous lines, offering to suppress oil backflow into the dispersed inlets given the viscosity ratio (oil to aqueous phase ≈ 10), and thereby stabilizing the flow‐focusing.

### 5.2 Microfabrication and Surface Treatment

The poly(dimethylsiloxane) (PDMS) part with microgrooves was fabricated using conventional soft lithography. SU‐8 3025 and SU‐8 3050 (Nippon Kayaku, Tokyo, Japan), which are negative photoresists, were spin‐coated onto a cut silicon wafer (3 cm × 7 cm) at 3000 rpm for 30 s to obtain a thickness of 25 and 50 µm, respectively. The prepared SU‐8‐coated wafers were prebaked on a hotplate at 95°C for 60 min, exposed to ultraviolet light through a film photomask (200 mJ cm^−2^), post‐exposure baked at 95°C for 3 min, and developed using 2‐acetoxy‐1‐methoxypropane to obtain the SU‐8 mold. To facilitate PDMS release, the mold surface was silanized by vapor‐phase chlorotrimethylsilane (Tokyo Chemical Industry, Tokyo, Japan) in a sealed dish and baked at 150°C.

The PDMS prepolymer and curing agent (Toray, Tokyo, Japan) were mixed at a 10: 1 weight ratio, degassed under vacuum for 30 min, poured into the mold, degassed again, and cured at 95°C for 40 min. The cured PDMS part was peeled off, and inlet/outlet holes (∼1 mm diameter) were created using a biopsy punch (Kai Industries, Gifu, Japan). The PDMS part and a glass slide were bonded by plasma treatment and baked at 120°C for 15 min.

After bonding, the microchannel surfaces were rendered hydrophobic to enable stable W/O droplet generation. For this treatment, fluorinated oil (HFE‐7500, 3M Novec Engineered fluid, MN, USA) containing 0.5v/v% trichloro(1H,1H,2H,2H‐perfluorooctyl) silane (Sigma–Aldrich, MO, USA) was introduced into the channels, followed by vacuum exposure for 30 min and baking at 120°C for 30 min. This fluorosilane treatment facilitates W/O emulsification and reduces wall wetting of the aqueous phase.

### 5.3 Preparation of DNA‐Functionalized Gold Nanoparticles

Gold nanoparticles (AuNPs, Nanopartz) with a nominal diameter of 10 nm were functionalized with thiolated single‐stranded DNA. Two types of DNA‐functionalized AuNPs were prepared: DNA‐AuNP A (DNA#1, 5′‐TCAACTATTCCTACCTACAAAAAAAAAAAAAAA‐S‐3′) and DNA‐AuNP B (DNA#2, 5′‐TCCACTCATACTCAGCAAAAAAAAAAAAAAAAA‐S‐3′), each containing a spacer region (poly(A)) and a complementary sequence. Two linker strands mediated hybridization between DNA‐AuNP A and DNA‐AuNP B: Linker #3 (DNA#3, 5′‐GTAGGTAGGAATAGTTGAAAATTCCTT‐3′) and Linker #4 (DNA#4, 5′‐TTGCTGAGTATGAGTGGAAAAAAGGAA‐3′), which hybridized with the complementary regions of DNA#1 and DNA#2 to form a stable assembly.

DNA conjugation to AuNPs followed a salt‐aging protocol to promote thiol–gold immobilization while minimizing nanoparticle aggregation. Briefly, AuNPs and thiolated ssDNA were mixed at a 1:400 molar ratio (AuNP: DNA) in 1× borate buffer (50 mmol L^−1^, pH 8.5). NaCl concentration was gradually increased from 0.05 to 0.5 mol L^−1^ in incremental steps; each step involved brief sonication (10 s) followed by 30 min of gentle rotation. After the final step, conjugates were washed four times by centrifugation (9300 rpm for 100 min) and resuspended in Phosphate buffer (Na_2_HPO_4_ 46.5 mmol L^−1^, KH_2_PO_4_ 19.8 mmol L^−1^, pH 7.2) to remove excess DNA. DNA–AuNP conjugate concentration was quantified by UV–vis spectroscopy (JASCO V‐630BIO).

### 5.4 Droplet Generation

Two aqueous solutions were prepared as dispersed phases. The first contained sodium chloride (1 mol L^−1^, Promega, Tokyo, Japan), phosphate (2/15 mol L^−1^, pH 7.2, Wako Pure Chemical Industries, Osaka, Japan), and two linker DNAs (Linker #3 and Linker #4, each at 10 µmol L^−1^). The second contained dNTPs (1.2 mmol L^−1^, Tokyo, Japan) and two types of DNA–NP conjugated (DNA‐AuNP A and DNA‐AuNP B, each at 100 nmol L^−1^). The continuous phase consisted of Isopropyl palmitate (IPP; Tokyo Chemical Industry, Tokyo, Japan) containing a surfactant (7 w/w%, ABIL EM 180, EVONIK, Germany), selected for its thermal stability (at 65°C) [32]. All solutions were handled using DNA LoBind Tubes (Eppendorf) and low‐retention pipette tips to minimize DNA adsorption.

### 5.5 Peripheral Equipment and Procedure

The fluidic reservoirs—a 15 mL centrifuge tube for the continuous phase and two 2 mL DNA LoBind microtubes for the dispersed phases—were each connected to the system through two independent lines. The headspace of every reservoir was coupled to a pressure regulator (Flow EZ, Fluigent, Le Kremlin‐Bicêtre, France) via polyurethane (PU) tubing (inner diameter 2.5 mm, outer diameter 4.0 mm), which provided the driving pressure. In parallel, the liquid outlet of each reservoir was plumbed to the corresponding device inlet via polytetrafluoroethylene (PTFE) tubing (inner diameter 0.5 mm, outer diameter 1.5 mm); an inline flow meter was placed in this PTFE delivery line between the reservoir and the device inlet to monitor the actual flow rate. In operation, pressure applied to the reservoir headspace through the PU line infused the liquid into the microchannel through the PTFE tube.

Droplet formation inside the microchannels was observed and recorded on an inverted optical microscope (ECLIPSE Ts2, Nikon, Tokyo, Japan) equipped with a high‐speed video camera (VW‐600 M, KEYENCE, Japan). The microfluidic device was mounted on a film heater (25.4 mm × 254 mm, KHA‐110, OMEGA Engineering Inc., CT, USA) under closed‐loop control (CN32PT‐445‐DC, OMEGA Engineering Inc., CT, USA) to maintain a setpoint of ∼65°C during emulsification and thereby prevent DNA‐NP aggregation inside the channels. After collection into microtubes, the emulsions were incubated from 55°C down to 35°C at a cooling rate of 0.01 °C min^−^^1^ in a benchtop incubator (IS402, Yamato Scientific Co., Ltd., Tokyo, Japan), ensuring slow, near‐equilibrium DNA hybridization and ordered crystal growth [33].

For control batch crystallization, linker and DNA‐NP solutions were mixed by pipetting in microtubes, sealed with Parafilm, equilibrated at 55°C (10 min), and cooled from 55°C to 35°C at 0.01 °C min^−^^1^. Precipitates were imaged on an upright microscope; crystal sizes were quantified as described below.

### 5.6 Measurement and Image Analysis Procedure

After incubation, crystallized DNA‐AuNPs within droplets were transferred into a glass‐bottom PDMS chamber (glass size: 24 mm × 60 mm, thickness 0.13–0.17 mm; chamber size: diameter 5 mm, height 5 mm) for imaging using an inverted optical microscope (TCS SP8 STED, Leica Microsystems GmbH, Wetzlar, Germany). To prevent evaporation during imaging at room temperature, a layer of oil was added to cover the droplets. Two focal planes were acquired for analysis: (i) the crystal plane and (ii) the droplet boundary, with rapid acquisition to minimize drift.

Droplet and crystal sizes were quantified from 2‐dimensional (2D) projections using image processing tools (ImageJ, NIH, NY, USA and MATLAB, The MathWorks, Inc., MA, USA). Droplet diameter (*D*_d_) was calculated from the projected droplet area (*A*_droplet_) as:

(2) $$D_{d}=2\sqrt{A_{\mathrm{droplet}}/\pi }$$

For crystals, single‐crystallite droplets were analyzed by contour extraction in ImageJ, while multi‐crystal droplets were processed in MATLAB with overlap handling. The crystal equivalent diameter (*D*_RD‐hex_​), based on a hexagonal projection of a rhombic dodecahedron (RD), was estimated as:

(3) $$D_{\mathrm{RD}-\mathrm{hex}}=2\sqrt{\frac{2A_{\mathrm{crystal}}}{3\sqrt{3}}}$$

The corresponding volume (*V*_RD‐hex_), representing the rhombic dodecahedron volume derived from the hexagonal projection, was calculated as:

(4) $$V_{\mathrm{RD}-\mathrm{hex}}=\frac{3\sqrt{6}}{16}{\left(\sqrt{\frac{8A_{\mathrm{crystal}}}{3\sqrt{3}}}\right)}^{3}$$

Sphere‐based estimates and the RD model derived from square projection yield smaller volumes compared to the RD model derived from hexagonal projection; therefore, *V*_RD‐hex_ was adopted as it better reflects the crystal morphology. Detailed derivations, geometric comparisons, and error analysis are provided in the Supporting Information (see ESI, Section S3).

### 5.7 Small‐Angle X‐Ray Scattering (SAXS) Analysis

SAXS measurements were performed at the bending‐magnet beamline BL40B2 of SPring‐8 using a PILATUS3 S 2 m area detector (DECTRIS Ltd, Baden, Switzerland) [34]. Suspensions of droplet‐grown DNA‐AuNP crystals were loaded into 1‐mm‐diameter quartz capillaries (Charles Supper Company Inc., Westborough, MA, USA) and measured at room temperature or at 45°C using a thermoelectric temperature‐controlled stage (TS62; Instec Inc., Boulder, CO, USA). The incident X‐ray wavelength (*λ*), sample‐to‐detector distance, beam size at the detector position, and X‐ray exposure time were 0.11 nm, 1.0 m, approximately 0.2 mm × 0.2 mm, and 0.3 or 3 s, respectively. Silver behenate (primary spacing *d*  =  5.838 nm) was measured for *q*‐calibration. Buffer‐only and oil/surfactant‐only controls were acquired and used for background subtraction.

SAXS data reduction followed established procedures [27]. Two‐dimensional scattering patterns were azimuthally integrated to obtain one‐dimensional intensity profiles *I*(*q*). The scattering vector was defined as *q* = (4π/*λ*) sin *θ*, where *θ* is half of the scattering angle [35]. To isolate interparticle correlations, the structure factor S(*q*) was calculated as S(*q*) = *I*_aggr, bg(_ *_q_*_)_/*I*_disp, bg(_ *_q_*_)_, where *I*_aggr, bg(_ *_q_*_)_ and *I*_disp, bg(_ *_q_*_)_ denote the background‐corrected one‐dimensional intensities for droplet‐grown DNA‐AuNP crystals and the dispersed DNA‐AuNPs, respectively.

## Author Contributions

Conceptualization: N.T. and M.T.; Methodology: N.T., M.T., S.S., and Y.Y.; Investigation: N.T., A.T., M.Y., L.Z., and M.T.; Visualization: N.T., A.T., and L.Z.; Funding acquisition: N.T., M.T., and Y.Y.; Supervision: N.T., M.T., S.S., and Y.Y.; Writing – original draft: N.T. and M.T.; Writing – review and editing: N.T., M.T., A.T., L.Z., M.Y., S.S., and Y.Y.

## Funding

This work was supported by Core Research for Evolutional Science and Technology (CREST), Japan Science and Technology Agency (JST) (grant number JPMJCR19S6), JSPS KAKENHI (grant numbers JP19H02555, JP25H00410, JP25K24703), and partially supported by JST Moonshot R&D Program (JPMJMS2217‐5‐1). Additional support was provided by the Tokuo Fujii Research Fund.

## Conflicts of Interest

The authors declare no conflict of interest.

## Supporting information




**Supporting File**: smll74787‐sup‐0001‐SuppMat.pdf.

## Data Availability

The data that support the findings of this study are available from the corresponding author upon reasonable request.
