# Supplementary material for: Thermally Regulated Droplet Microfluidics Enables Uniform, Size‐Controllable DNA‐Nanoparticle Superlattices
Source: Small. 2026 Aug 10;22(54):e74787. doi: 10.1002/smll.74787 (PMC13614272; doi:10.1002/smll.74787)
Supplement: Supplementary file 1 [file SMLL-22-e74787-s001.pdf]

Supporting Information

Thermally regulated droplet microfluidics enables uniform, size-controllable DNA-nanoparticle superlattices

Naotomo Tottori,[1*] *Miho Tagawa,*[2,3*] *Azusa Takao,*[1] *Lidong Zhang,*[3] *Maasa Yokomori,*[1†] *Shinya Sakuma,*[1] *and Yoko Yamanishi*[1]

[1] Department of Mechanical Engineering, Faculty of Engineering, Kyushu University, 744 Motooka, Nishi-ku, Fukuoka 819-0395, Japan.

[2] Division of Materials Research (DM), Institute of Materials and Systems for Sustainability (IMaSS), Nagoya University, Furo-cho, Chikusa-ku, Nagoya 464-8601, Japan

[3] Department of Materials Process Engineering, Nagoya University, Furo-cho, Chikusa-ku, Nagoya 464-8603, Japan

[†] Present address: Department of Precision Mechanical Engineering, School of Science and Engineering, Chuo University, 1-13-27, Kasuga, Bunkyo-ku, 112-8551, Tokyo, Japan.

*email: tottori@mech.kyushu-u.ac.jp

*email: tagawa.miho.z5@f.mail.nagoya-u.ac.jp

Section S1. Peak profile analysis of SAXS data

For the analysis of structural imperfections, we distinguished the influence of the crystallite size from the influence of the lattice distortion using SAXS profiles. Using the powder X-ray diffraction method, the Voigt function, pseudo-Voigt function, and Pearson VII function are generally used for profile functions. [1, 2] The pseudo-Voigt function is also used for the fitting of SAXS profiles. [3] Therefore, we adopted the pseudo-Voigt function, which is the sum of the Gaussian function and the Lorentzian function, as a peak profile function. The broadening of the peak width is attributable to instrument factors (X-ray beam divergence, energy bandwidth, and geometrical influence), single-crystal domain (crystallite) sizes of the superlattices, and mainly the distortion in the superlattices. In the present analysis, the Lorentzian component was primarily associated with crystallite size effects, whereas the Gaussian component was used as an indicator of lattice distortion together with instrumental broadening. The pseudo-Voigt function is as follows:

$$P(q) = A_{\mathrm{dif}}\left[\eta \frac{\sigma_{\mathrm{L}}/\pi}{\left(q - q_{\mathrm{hkl}}\right)^{2} + \sigma_{\mathrm{L}}{}^{2}} + (1-\eta)\frac{1}{\sqrt{2\pi\sigma_{\mathrm{G}}{}^{2}}}\exp\left(-\frac{\left(q\text{-}q_{\mathrm{hkl}}\right)^{2}}{2\sigma_{\mathrm{G}}{}^{2}}\right)\right] + B_{\mathrm{dif}} \qquad \text{(S1-1)}$$

where A_{dif} is the amplitude, q_{hkl} is the peak position, σ_{L} and σ_{G} are standard deviations, η is the ratio of the Gaussian to the Lorentzian function and B_{dif} is the background. The integral widths of the Lorentzian and Gaussian functions are represented as $\beta_{\mathrm{L}} = \pi\sigma_{\mathrm{L}}$ and $\beta_{\mathrm{G}} = \sqrt{2\pi}\sigma_{\mathrm{G}}$. However, it is challenging to uniquely determine fitting parameters without introducing model-dependent assumptions.

The peak broadening attributed to the crystallite size (β_{size}) was approximately estimated using the following Scherrer's equation.

$$\beta_{\mathrm{size}} = \frac{2\pi K}{\tau_{\mathrm{hkl}}} \qquad \text{(S1-2)}$$

Here, K is the Scherrer constant, and τ_{hkl} is the length in the [hkl] direction, which is of the 1D crystallite size. Ignoring the crystallite shape and [hkl] dependence, the Scherrer constant K is generally 1 when the peak width is equal to the integral width of the diffraction peak.

The peak width of the Lorentzian function is assumed to be approximately equal to the value calculated using Scherrer's equation ($\beta_{\mathrm{L}} \approx \beta_{\mathrm{size}}$). We fitted the first peak of the structure factor in such a manner that the peak width of the Lorentzian function was approximately equal to the estimated value. From the above equation, we estimated the parameters q_{hkl}, σ_{L}, and σ_{G}. Furthermore, when the integral widths of the diffraction peaks were greater than 0.05 nm^{-1} during this study, the g-factor did not change by 0.01, even if the crystallite size estimation deviated by a factor of two.

Next, we considered that the instrument factor was derived from the broadening of the X-ray and detector errors. The peak width attributed to the instrument factor β_{inst} contributes mainly to the determination of three values.

$$\beta_{inst}^2 = \beta_{geo}^2 + \beta_{BW}^2 + \beta_{div}^2 \quad \text{(S1-3)}$$

Here, β_{geo}, β_{BW}, β_{div} are the widths contributing to the geometry, energy bandwidth, and X-ray beam divergence, respectively. In this study, the beam divergence is negligible as the divergence angle is small.

$$\beta_{geo} = \frac{2\pi}{\lambda}\frac{w}{L} tan 2\theta_{hkl} \approx \frac{4\pi}{\lambda}\frac{w}{L_D}\theta_{hkl} \quad \text{(S1-4)}$$

$$\beta_{BW} = \frac{2\pi}{\lambda}\frac{2\Delta E}{E} tan\theta_{hkl} \approx \frac{4\pi}{\lambda}\frac{\Delta E}{E}\theta_{hkl} \quad \text{(S1-5)}$$

Here, λ is the X-ray wavelength, w is the X-ray spot diameter on a detector, L_D is the sample-to-detector distance, θ_{hkl} is the diffraction angle, and ΔE / E is the energy bandwidth. In this study, the wavelength was 0.11 nm, the sample-to-detector distance was 4 m, the dimensions at half maximum of the spot diameter were approximately 0.2 mm × 0.2 mm at the detector position, and the energy bandwidth was approximately 10^{-4}. The instrument factor is assumed to be a Gaussian function. The integral width attributed to lattice distortion β_{dist} can be determined as follows: $\beta_{dist}^2 = \beta_G^2 - \beta_{inst}^2$.

References

1. T. Ida, M. Ando, H. Toraya, "Extended pseudo-voigt function for approximating the voigt profile," *J. Appl. Cryst.* **2000**, 33, 1311–1316.
2. D. Balzar, "X-ray diffraction line broadening: Modeling and applications to high-Tc superconductors," *J. Res. Natl. Inst. Stand. Technol.* **1993**, 98, 321–353.
3. Y. Zhang, F. Lu, K. G. Yager, D. van der Lelie, O. Gang, "A general strategy for the DNA-mediated self-assembly of functional nanoparticles into heterogeneous systems," *Nat. Nanotechnol.* **2013**, 8, 865–872.

Section S2. Size-dependent nucleation and growth dynamics under confinement

The transition from predominantly single-crystallite objects in small droplets (~9–23 μm) to multiple crystals in large droplets (> 23 μm) (Figure 3B) can be described in terms of the size dependence of stochastic nucleation probabilities within a stochastic nucleation framework grounded in Classical Nucleation Theory (CNT). The homogeneous nucleation rate is given as follows:

$$J(t) = J_0 \exp\left(-\frac{\Delta G^*(t)}{k_B T}\right), \Delta G^* \propto \frac{\gamma^3}{(\Delta\mu)^2} \quad \text{(S2-1)}$$

where J_0 is a kinetic prefactor, ΔG^* is the critical free-energy barrier, γ is the effective interfacial energy, and $\Delta\mu$ is the chemical potential driving supersaturation, k_B is Boltzmann's constant, and T is the absolute temperature.[1–3] As commonly applied in droplet-based nucleation studies,[2, 3] the probability of at least one nucleation event during the supersaturation window t_{nuct} for $V_{droplet}$ is calculated as:

$$P(\geq 1) = 1 - \exp\left(-\int_0^{t_{nuc}} J(t) V_{droplet} \mathrm{d}t\right) \quad \text{(S2-2)}$$

This volume–time integral highlights operative design handles: increasing $V_{droplet}$ or widening t_{nuc} (e.g., via faster cooling that rapidly ramps $\Delta\mu$) increases the likelihood of multiple, statistically independent nucleation events. Under ultra-slow cooling, the supersaturation increases gently, so the instantaneous nucleation rate remains modest up to the first event; once the first nucleus appears, its growth rapidly depletes supersaturation throughout the small droplet (fast diffusion), collapsing $J(t)$ and effectively truncating the high-J window for parallel nucleation. This statistical suppression of parallel nucleation increases the probability that crystallization proceeds from a single dominant nucleus in smaller droplets, where convective recirculation is weak, and supersaturation fields remain spatially uniform. However, while CNT provides a useful baseline for the thermodynamic barrier term ΔG^*, DNA-NP crystallization can deviate from the diffusion-limited kinetic prefactor often assumed in CNT because friction-limited interfacial rearrangements of DNA linkages slow the attainment of coherent crystal interfaces.[4] To capture this effect conceptually, we introduce a simplified modification to the kinetic prefactor:

$$J_0^{(eff)} = \frac{J_0}{1+\tau_{rearr}/\tau_{attach}} = J_0 \frac{\tau_{attach}}{\tau_{attach}+\tau_{rearr}} \quad \text{(S2-3)}$$

This expression is not derived from first principles but serves as a conceptual representation of friction-limited kinetics, inspired by the rolling-limited attachment model proposed by Hensley et al.[4] We note that Hensley et al.[4] introduces an explicit intermediate-state kinetic scheme

(an adsorbed, non-lattice-registered state) and shows that "rolling/sliding"-limited attachment reduces the effective prefactor relative to a diffusion-limited assumption; Eq. (S2-3) here is a compact representation of that qualitative effect rather than a full reproduction of the intermediate-state rate equations. Here, τ_{attach} is the timescale for bond formation, and τ_{rearr} represents additional interfacial rearrangements necessary for defect-minimized growth. Larger τ_{rearr} effectively reduces $J_0^{(eff)}$, narrowing the practical nucleation window in which parallel nucleation is statistically disfavored, relative to a diffusion-limited CNT prefactor. While we do not directly tune τ_{rearr} in this study, chemistry-based handles (temperature, ionic strength, DNA graft density, and spacer design) can modulate rearrangement-limited kinetics; when used in concert with droplet size, these handles expand the single-crystal domain by lowering the effective peak of $J(t)$ prior to the first event and truncating the high-J window thereafter. Complementarily, confinement reduces hydrodynamic recirculation and suppresses Marangoni-driven and solutal fluctuations, thereby truncating the high-J window and reducing the probability of multiple, concurrent nucleation events.

Experimentally, multiplets remained rare up to ~23 μm but appeared more frequently around ~27 μm, consistent with Figure 3B, though 2D projection may underestimate multiplicity for larger droplets. Together with the near-linear $V_{crystal}$–$V_{droplet}$ scaling (Figure 4), these observations provide a practical basis for tuning crystallization outcomes by statistically biasing the probability of parallel nucleation through droplet-size control, without assuming a direct one-to-one correspondence between nucleation events and final crystals. These insights align with prior droplet-based CNT frameworks and extend them by incorporating friction-limited interfacial rearrangements critical for DNA-NP systems.

References

1. D. Kashchiev, *Nucleation: Basic Theory with Applications*. Elsevier, **2000**.
2. P. Laval, J. B. Salmon, and M. Joanicot, "A microfluidic device for investigating crystal nucleation kinetics," *J. Cryst. Growth* **2007**, 303, 622–628.
3. R. D. Dombrowski, J. D. Litster, N. J. Wagner, and Y. He, "Crystallization of alpha-lactose monohydrate in a drop-based microfluidic crystallizer," *Chem. Eng. Sci.* **2007**, 62, 4802–4810.
4. A. Hensley, W. M. Jacobs, and W. B. Rogers, "Self-assembly of photonic crystals by controlling the nucleation and growth of DNA-coated colloids," *Proc. Natl. Acad. Sci.* **2022**, 119, e2114050118.

Section S3. Geometric approximations for crystal volume estimation

To evaluate the effect of geometric assumptions on volume estimation, crystal volumes were calculated using three models based on the projected area (A_{crystal}):

Crystal volume calculated by sphere-equivalent model (V_{sphere}):

$$V_{\mathrm{sphere}} = \frac{4}{3}\pi\left(\sqrt{\frac{A_{\mathrm{crystal}}}{\pi}}\right)^3 \tag{S3-1}$$

Crystal volume calculated by rhombic dodecahedron (RD) model derived from square projection ($V_{\mathrm{RD\text{-}sq}}$) :

$$V_{\mathrm{RD\text{-}sq}} = \frac{\sqrt{2}}{2}\left(\sqrt{A_{\mathrm{crystal}}}\right)^3 \tag{S3-2}$$

Crystal volume calculated by Rhombic dodecahedron model derived from hexagonal projection ($V_{\mathrm{RD\text{-}hex}}$) (used in the main text):

$$V_{\mathrm{RD\text{-}hex}} = \frac{3\sqrt{6}}{16}\left(\sqrt{\frac{8A_{\mathrm{crystal}}}{3\sqrt{3}}}\right)^3 \tag{S3-3}$$

Sphere-based estimates and the RD model derived from square projection yield smaller volumes than the RD model derived from hexagonal projection for the same projected area. RD-based values provide a more realistic approximation for DNA-NP crystals because the rhombic dodecahedron closely represents the actual crystal morphology. Among RD projections, the hexagonal view was selected because it occurs more frequently than the square view due to the symmetry of the RD structure, making it geometrically more representative.

Supplementary Figures

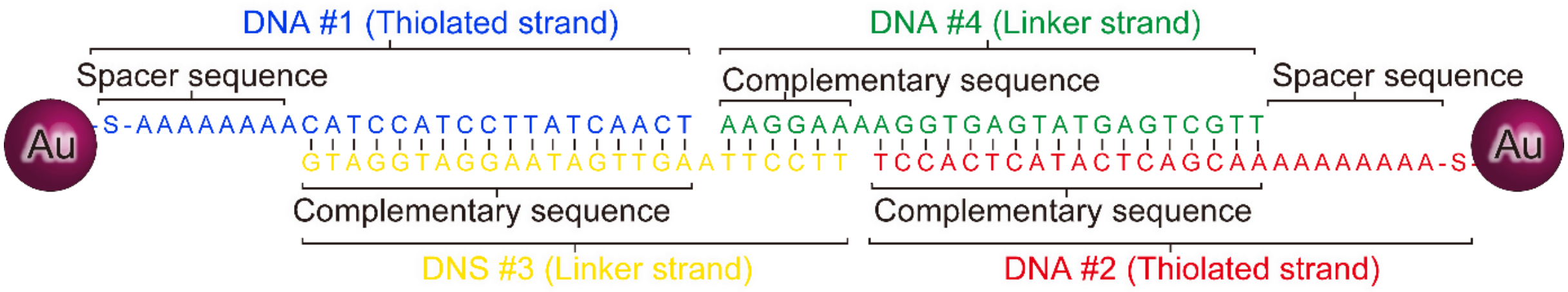

Figure S1. Schematic illustration of the experimental design showing DNA-mediated linkage between DNA-functionalized gold nanoparticles (DNA-AuNPs). This example represents a binary-component, non-self-complementary system. For clarity, only a single interparticle linkage is depicted, and the diagram is not drawn to scale. A = adenine; T = thymine; C = cytosine; G = guanine; S = thiol group at the DNA terminus. Thiolated DNA strands #1 and #2 each contain eight spacer bases and eighteen recognition bases that hybridize with complementary regions in linker DNA strands #3 and #4, respectively. Linker DNA strand #3 further hybridizes with strand #4 through six complementary bases. DNA-AuNP assemblies are expected to form body-centered cubic (bcc) superlattices.

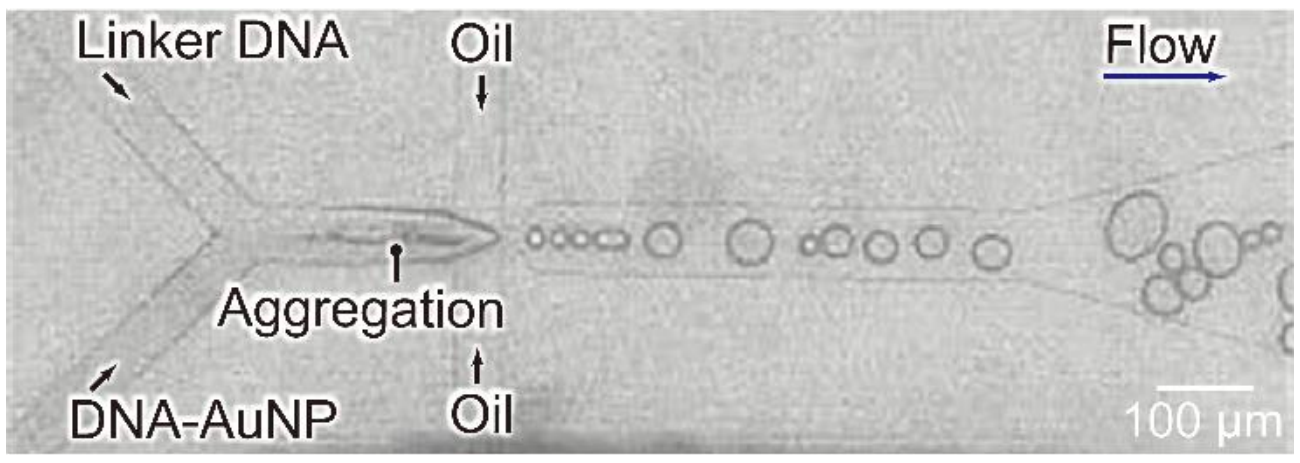

Figure S2. Effect of heating on droplet formation. Droplet formation at the flow-focusing junction under a no-heating condition (contrast with Fig. 2A showing a heated condition).

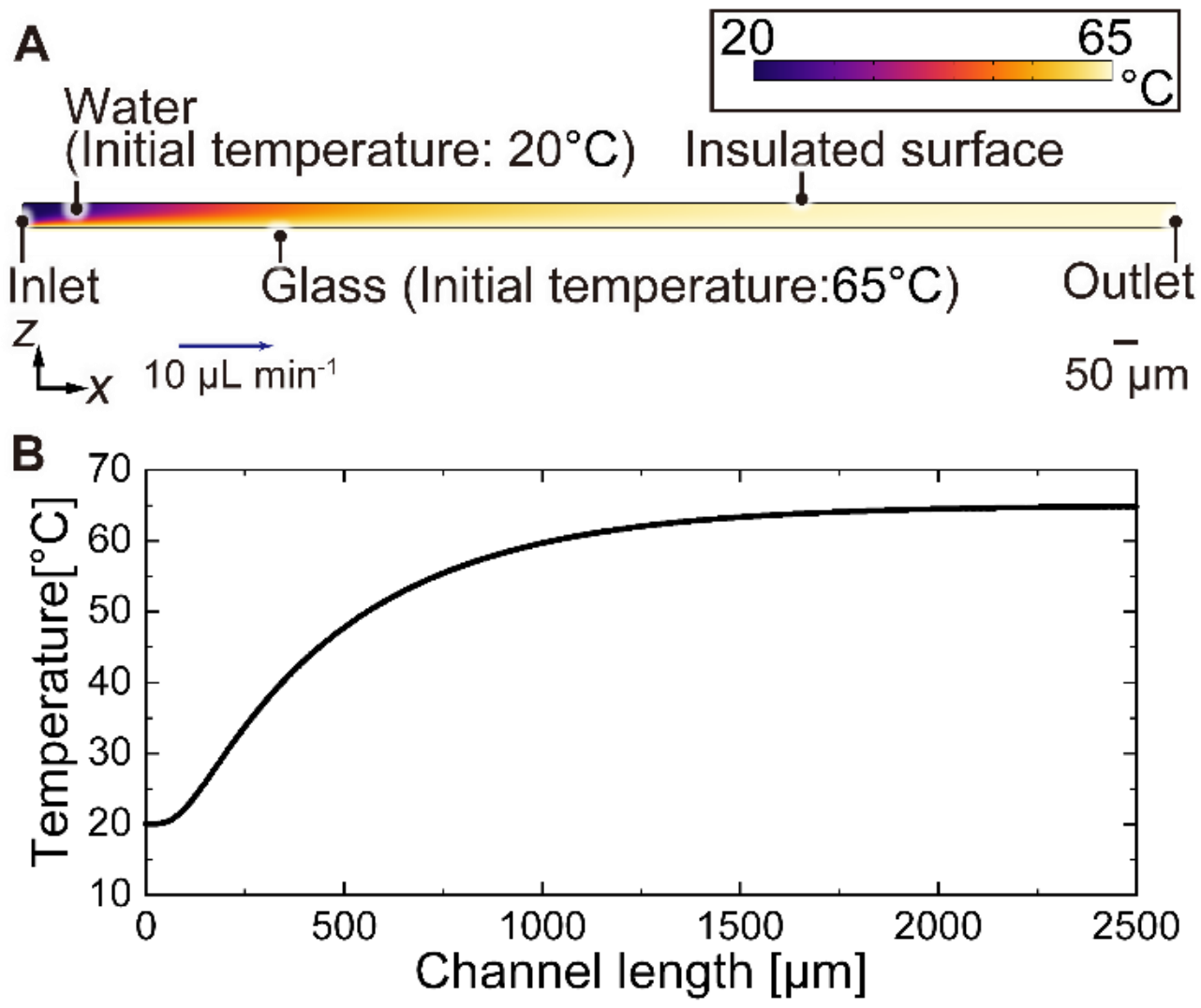

Figure S3. Simulation of dispersed-phase heating in a serpentine microchannel. (A) Temperature distribution along the serpentine channel simulated using COMSOL Multiphysics (COMSOL Multiphysics v6.1a, COMSOL AB, Stockholm, Sweden) by solving Navier–Stokes and transient heat equations in a 2D channel section (50 μm height, 2500 μm length). The bottom wall was set to 65°C, and other boundaries were adiabatic. For water at 20°C and Q = 10 μL min^{-1}, the centerline fluid reached 64.5°C after approximately 2.02 mm. (B) Temperature profile at the top surface as a function of channel length.

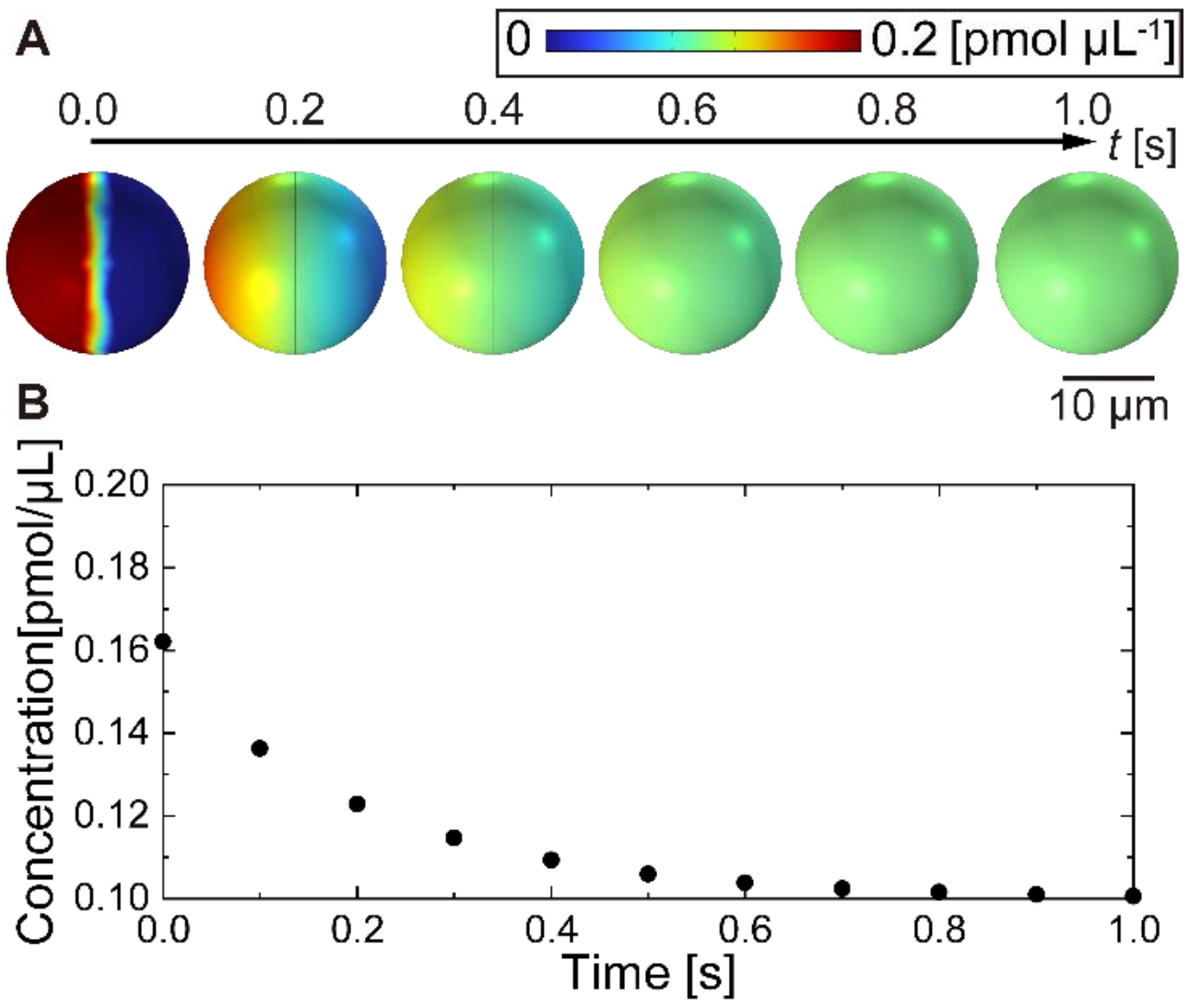

Figure S4. Simulation of mixing of solutions in a microdroplet. (A) Time evolution of concentration distribution inside a spherical droplet (diameter: 20 μm) simulated using a diffusion-only model. The droplet was initially half-filled with DNA-NP solution (0.2 pmol μL^{-1}) and half water. (B) Temporal change in average concentration within the DNA-NP-containing hemisphere, showing a decrease from 0.2 pmol μL^{-1} toward the equilibrium value of 0.1 pmol μL^{-1}. The model predicted that the solutions became effectively mixed within < 1 s for 10 nm particles at 338 K ($D \approx 1.14\times10^{-10}$ m^2 s^{-1}), confirming that diffusion alone is sufficient to achieve effective homogenization under the experimental conditions.
